# Factors affecting future specialty choice among medical students in Kuwait

**DOI:** 10.3402/meo.v17i0.19587

**Published:** 2012-12-20

**Authors:** Rawan Al-Fouzan, Sarah Al-Ajlan, Yousef Marwan, Mervat Al-Saleh

**Affiliations:** Department of Surgery, Faculty of Medicine, Health Science Center, Kuwait University, Al-Jabriya, Kuwait

**Keywords:** medical education, future career, specialty choice, career choice, residency, Kuwait

## Abstract

**Background:**

Choosing a medical specialty can be either a daunting and confusing experience for some medical students and junior doctors or a foregone conclusion to others. The aim of this study is to evaluate factors affecting future specialty choice among medical students in Kuwait University.

**Methods:**

A self-administered questionnaire was used to collect data from medical students registered in Kuwait University during the academic year 2011/2012. Chi-square test and logistic regression were used to test the association between deciding a future specialty and students’ sociodemographic and academic factors.

**Results:**

Of the 422 students approached, 387 (91.7%) decided to participate. A total of 144 (37.2%) students made a decision regarding their choice of future medical specialty. Pediatrics, general surgery, and cardiology were the most desired specialties – 18 (12.5%), 17 (11.8%), and 16 (11.1%) students requested these specialties, respectively. Only 61 (42.4%) of those who selected a future specialty received advice regarding their choice. Looking for a good treatment outcome for patients (66; 45.8%) and a challenging specialty (58; 40.3%) were the most influencing incentives when selecting a future specialty. Students in the clinical phase of their study were 3.014 (95% CI: 1.498–6.065) more likely to report on their decision regarding a future specialty compared to students in the basic medical sciences phase (*p*=0.002).

**Conclusion:**

A variety of factors appeared to inspire medical students in Kuwait to choose a future medical specialty. When identified, these factors can be used by mentors of medical students and directors of residency training programs to motivate students to choose specialties that are limited in Kuwait.

## Introduction

Choosing a medical specialty can be either a daunting and confusing experience for some medical students and junior doctors, or a foregone conclusion to others. Many factors contribute to one's decision with regards to motivation and reason to choose a specific medical specialty. Whether one chooses a specific specialty due to advice from friends or family, admiration of a certain mentor or genuine interest in the specialty, there are many incentives and factors that converge to result in the final decision (1–5). While motivations do vary according to specialties, they may include lifestyle choices, a possibility of private practice, an interest in specific diseases, a varied scope of practice, an interest in research and teaching, or to gain a higher income (2, 4, 6–8).

Medical graduates who are Kuwait’ nationals can either join residency programs in Kuwait (though not all specialties are available) or be sponsored to join a residency program abroad such as North America, Europe, or Saudi Arabia. Alternatively, medical graduates who do not hold Kuwait's nationality can join the healthcare system of Kuwait after they complete their training outside Kuwait. This depends on the need of the doctor's chosen specialty at the time of application. Also, they can try to apply for residency training programs available in Kuwait; however, the priority is usually given to applicants with Kuwaiti citizenship.

It is imperative to realize the importance of future specialty choices and the impact these decisions have on the physician workforce in Kuwait. As an objective comparison, there were a total of 3,029 physicians in Kuwait in 1994, which rose to 5,293 physicians by 2006. Although this is a substantial increase over 12 years in general, the number of physicians per 1,000 of population in 1994 was 23.8 per 1,000, which dropped to 15.6 per 1,000 in 2006 (9). In 2011, the total number of physicians in Kuwait increased to 5,680 (10). This raises the question of whether the workforce is able to meet the demands of patients on a national level, considering the constant population growth, and subsequently, if specialties which are available in Kuwait are able to meet not only the demands of the population but also the desires of junior doctors.

It is clear that as a medical community in Kuwait, the possibilities of new specialties and future prospects are under constant transformation and upheaval due to a variety of internal and external factors. Therefore, it is important to study the dynamics which affect students as individuals. Hence, we conducted this study to understand the reasons for selecting a future specialty among medical students in Kuwait and to determine if these factors vary according to the student's sociodemographic or academic characteristics.

## Methods

### Data collection instrument

After a comprehensive review of relevant topics in the literature, we developed a self-administered questionnaire in English consisting of 16 questions divided into two main sections. Section I consists of a set of 10 questions about the sociodemographic and academic characteristics including age, gender, nationality, marital status, monthly family income, governorate, parents education, year of study in medical school, and grade point average (GPA). All questions of section I were closed ended except for age and GPA. Section II included questions about the choice of future specialty, sources of advice for choosing a specialty, and reasons for selecting a specific specialty. The question about the choice of future specialty was open ended, and the question regarding the reasons for choosing this specialty consisted of 22 choices of possible reasons with an extra choice to specify reasons other than those proposed in our questionnaire. The rest of the questions of section II were closed ended. The questionnaire was pretested on 26 randomly selected students to ensure clarity of the questions.

### Study design and participants

This cross-sectional study was conducted in October 2011. Our study sample consisted of students registered in the Faculty of Medicine, Kuwait University (FOM, KU), during the academic year 2011/2012. Thus, all students, with the exception of 1st year students, were eligible for inclusion in the study. The 1st year students were not included as they are not considered medical students yet (i.e., 1st year students are considered health sciences students; once they finish the academic year they need to decide whether they would like to continue their studies in Medicine or in other health sciences such as Dentistry and Pharmacy). We were able to contact 422 (70%) students, out of 606 students registered in FOM, KU. All students were invited to participate in the study without considering their grades or any other factors. Only 35 (8.3%) students declined to participate in the study without citing any reason.

After explaining the study objectives, a written informed consent was obtained from each participant. Also, the participants were assured of confidentiality of the collected information and that they were free to decline participation in the study. The study protocol and data collection instrument was approved by the Joint Ethics Committee of the Ministry of Health in Kuwait and FOM, KU. Permission to use the questionnaire for the study sample was obtained from the administration of the FOM, KU.

### Statistical analysis

We analyzed data using the Statistical Package for Social Sciences (SPSS) with a two-tailed *p*-value <5%, which is considered as the cut-off value for significance. We entered the data and then computed frequencies and percentages of all variables. In addition, Chi-square test was used to assess the association between deciding a future specialty (outcome variable) and the sociodemographic and academic characteristics of the participants. This test was used because we were assessing the association between qualitative variables, and our outcome variable was binary. Moreover, multivariate analysis using logistic regression was conducted. The dependent variable in the regression model was deciding a future specialty, which was binary, and the independent variables were age, gender, nationality, marital status, monthly family income, level of study in medical school, and GPA. Level of study in medical school was included because it was found to be significant in the crude analyses. Other variables were included in the regression model because they were either important in our sample, or because they were found to be associated with choosing a future specialty in previous studies. Level of study in medical school was considered the most important associated factor. Other variables were added one at a time to ascertain if they were independently associated with choosing a future specialty after controlling for the level of study in medical school.

## Results

Out of 387 medical students in Kuwait University, future specialty choices and the influence of these choices have been evaluated.

In Table 1, sociodemographic characteristics of the study population are illustrated. The age of the participants ranged from 17 to 25 years, with 194 (50.1%) participants aged 22 years. Three-hundred and thirty four (86.3%) students were Kuwaiti. Female students accounted for 221 (57.1%), while male students were 166 (42.9%). Most of our participants were unmarried (346; 89.4%). A monthly family income of more than 2,000 Kuwait Dinars (KD; 1 KD=3.5 US dollars) was reported by 237 (61%) participants. In terms of parents’ education, 252 (65.1%) fathers and 247 (63.8%) mothers of our participants obtained a bachelor degree or higher educational degrees. The highest proportion (150; 38.8%) of students lived in Hawalli governorate, while the minority (16; 4.1%) lived in Al-Jahra governorate. Two-hundred and twelve (54.8%) participants were in their clinical years of study, while the rest (175; 45.2%) were in their basic medical sciences phase of study. In terms of academic performance, 86 (29.3%) students reported a GPA of more than 3.0 (out of 4.0), while 106 (36.2%) reported a GPA of less than 2.5. Two hundred and forty three (62.8%) students had not decided on a future specialty yet.

**Table 1:** Distribution of the sociodemographic characteristics of medical students in Kuwait University, academic year 2011/2012

Characteristics	*n*	%
Total	387	100.0
Age (years)		
<22	194	50.1
≥22	193	49.9
*Mean±SD*	*21.45±1.715*	
Range	17–25	
Nationality		
Kuwaiti	334	86.3
Non-Kuwaiti	53	13.7
Gender		
Male	166	42.9
Female	221	57.1
Marital status		
Married	41	10.6
Not married	346	89.4
Monthly family income		
≤2000 KD	150	38.8
>2000 KD	237	61.2
Father's education		
High school or less	68	17.6
Diploma	67	17.3
Bachelor	139	35.9
Master or PhD	113	29.2
Mother's education		
High school or less	59	15.2
Diploma	81	20.9
Bachelor	177	45.7
Master or PhD	70	18.1
Governorate		
Al-Ahmadi	20	5.2
Al-Asimah	107	27.6
Al-Farwaniya	40	10.3
Al-Jahra	16	4.1
Hawalli	150	38.8
Mubarak Al-Kabeer	54	14.0
Level of study		
Basic medical sciences	175	45.2
Clinical sciences	212	54.8
GPA^a^		
≤2.5/4.0	106	36.2
2.51/4.0 – 2.99/4.0	101	34.5
≥3.0/4.0	86	29.3
Selected a specialty		
No	243	62.8
Yes	144	37.2

SD=standard deviation; KD=Kuwait dinars; GPA=grade point average.

a Total number is not 387 because of missing values.

**Fig. 1:**
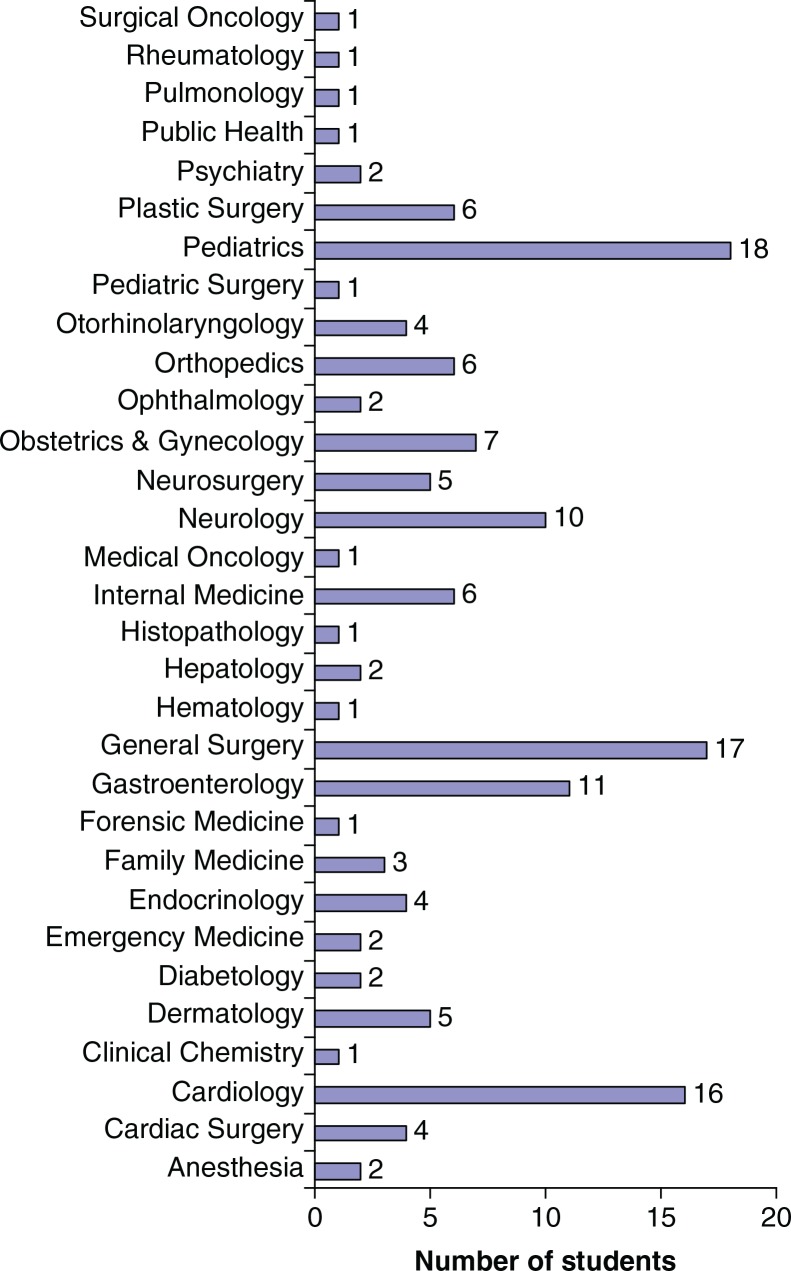
Future specialty choices of medical students in Kuwait University, academic year 2011/2012.

Figure 1 demonstrates the future specialty choices of our participants. The three most desired specialties are pediatrics, general surgery, and cardiology [18 (12.5%), 17 (11.8%), and 16 (11.1%) students, respectively]. No other specialties other than those mentioned in Fig. 1 were desired by our sample of medical students of Kuwait University.

The sources of advice to choose a future medical specialty among our participants are outlined in Table 2. Among the students who have chosen their future specialty, 61 (42.4%) received advice regarding specialty choice. The sources of advice varied: 24 (16.7%) students received advice from relatives, 16 (11.1%) from their friends, 17 (11.8%) from faculty staff, and 39 (27.1%) from practicing doctors.

**Table 2:** Source of advice for choosing a future specialty among medical students in Kuwait University, academic year 2011/2012

Received advice for future specialty	*n*	%
No	83	57.6
Yes	61	42.4
Source of advice^a^		
Family	24	16.7
Friends	16	11.1
Faculty staff	17	11.8
Practicing doctors	39	27.1

a Include only frequency and percentage of participants who selected ‘Yes’.

The reasons behind choosing a future specialty are shown in Table 3. The most frequently cited reason for choosing a future specialty was the preference to see good treatment outcomes for patients (66; 45.8%), followed by the desire to work in a challenging specialty (58; 40.3%). Conversely, only 10 (6.9%) students reported that a residency program of short duration was a reason to choose their future specialty.

**Table 3:** Reasons of choosing a future specialty among medical students in Kuwait University, academic year 2011/2012

Rank	Reasons of choosing a specialty^a^	*n*	%
1	I would like to see good treatment outcomes on my patients	66	45.8
2	I'm looking for a challenging specialty	58	40.3
3/4	I'm looking for specialty with good reputation and prestige	44	30.6
3/4	I want to have a good social life	44	30.6
5	Lack of specialist in this specialty in my country	43	29.9
6	I want a high income	40	27.8
7	I would like to have a long-term relationship with my patients	33	22.9
8/9/10/11	I'm trying to become like a doctor known to me	32	22.2
8/9/10/11	I will have better opportunities in the private sector	32	22.2
8/9/10/11	I had a personal experience that stimulated my interest in this specialty (me/family member had a condition related to the specialty stimulated my interest)	32	22.2
8/9/10/11	I'm looking for a specialty with acceptable working hours	32	22.2
12	I would like to see a wide variety of patients with different conditions	31	21.5
13	The specialty I want offers more research opportunities	28	19.4
14/15	I'm looking for a specialty with acceptable on-call duty	25	17.4
14/15	I prefer to treat emergency cases	25	17.4
16/17	I prefer to treat non-urgent cases	22	15.3
16/17	I would like to see a narrow group of patients with specific problems	22	15.3
18/19	I would like to focus on treating patients in the ward (inpatients)	21	14.6
18/19	I would like to focus on treating patients in clinics (outpatients)	21	14.6
20/21	I do not want to have a direct interaction with patients	14	9.7
20/21	I want to treat less complicated patients	14	9.7
22	I'm looking for a specialty program with a short duration	10	6.9
	Other reasons^b^	22	15.3

a Include only frequency and percentage of participants who selected ‘Yes’.

b Other reasons include: (1) Prefer an interventional medical specialty. (2) Prefer a specialty that involves treating medical and surgical conditions. (3) Prefer to work with children. (4) Work better under stress. (5) Enjoyed the specialty during my study. (6) Special interest in specific diseases.

The Chi-square analysis revealed that the level of study in medical school was significantly associated with deciding on a future medical specialty (*p*=0.011): 91 (42.9%) students in their clinical phase of study decided a future specialty compared to 53 (30.3%) students in their basic sciences phase of study. This was confirmed by the multivariate logistic regression model (Table 4), where we found that students in their clinical phase of study were 3.014 (95% CI: 1.498–6.065) more likely to decide a future medical specialty than students in their basic sciences phase of study (*p*=0.002). None of the other variables were significantly associated with deciding a future specialty among the participants.

**Table 4:** Logistic regression of choosing a future specialty among medical students in Kuwait University, academic year 2011/2012

		Univariate logistic regression		Multivariate logistic regression	
			
Characteristics	Subgroup *n*	Crude OR (95% CI)	*p*	Adjusted OR (95% CI)	*p*
Age (years)					
<22	194	Reference		Reference	
≥22	193	1.204 (0.797–1.819)	0.379	0.580 (0.293–1.150)	0.119
Gender					
Female	221	Reference		Reference	
Male	166	1.157 (0.763–1.753)	0.492	1.215 (0.791–1.866)	0.373
Nationality					
Non-Kuwaiti	53	Reference		Reference	
Kuwaiti	334	0.811 (0.449–1.463)	0.486	0.934 (0.501–1.739)	0.829
Marital status					
Not married	346	Reference		Reference	
Married	41	0.970 (0.496–1.900)	0.930	0.810 (0.389–1.690)	0.575
Monthly family income					
≤2000 KD	150	Reference		Reference	
>2000 KD	237	1.088 (0.712–1.664)	0.695	1.004 (0.637–1.582)	0.987
Level of study					
Basic medical sciences	175	Reference		Reference	
Clinical sciences	212	1.731 (1.135–2.640)	0.011	3.014 (1.498–6.065)	0.002
Grade point average					
≤2.5	106	Reference		Reference	
2.51–2.99	101	1.332 (0.752–2.359)	0.326	1.496 (0.833–2.686)	0.178
≥3.0	86	1.379 (0.832–2.287)	0.213	1.606 (0.940–2.744)	0.083

OR=odds ratio; KD=Kuwait dinars.

## Discussion and conclusions

Although this study is based on a single survey in the Faculty of Medicine, it has been informative in terms of revealing the myriad of factors affecting medical students’ decision to choose a certain specialty in Kuwait, starting from the earlier years of study and building up toward graduation; arguably a time in which students are expected to reach a clearer, if not definitive view of their future career path.

Our results revealed that students in later years of study (i.e., clinical years) were more likely to report that they had chosen a future specialty. This difference is probably due to exposure to more specialties at later stages. In the University of Glasgow, exposure to general practice influenced medical student to choose this specialty for their future medical career (11). This indicates that during the progression in medical school, students’ choices of future specialty might change.

The minority that had chosen a specialty tended to sway toward pediatrics, general surgery, cardiology, neurology, and gastroenterology. A similar survey of medical students in the nearby Jordan University of Science and Technology showed that surgery, internal medicine, pediatrics, and obstetrics and gynecology were the most preferred specialty among medical students studying there (12). Similarly, a study of Canadian medical students showed that the most popular choices among students were internal medicine and medical subspecialties, surgery and surgical subspecialties, pediatrics, and family medicine (13). In Japan, internal medicine, general surgery, pediatrics, and emergency medicine were the specialties of most interest to medical students at the Yamaguchi University School of Medicine (14). This indicates a possible trend of specialty choices among medical students, which influences the number of physicians in different specialties. Therefore, it may be due to the fact that these are the specialties that the students are in touch with on a daily basis. This is supported in a study of the distribution of Kuwaiti physicians over the period 1968–1999, which shows that the majority of graduates attained their specialty at medicine, followed by pediatrics, family medicine, obstetrics and gynecology, and surgery followed by other specialties (15).

Surprisingly, the majority (243; 62.8%) of students in our study had not chosen a specialty at all, many of whom have been exposed to a wide variety of specialties. By the time they have reached their final year, most students rotated through all major medical specialties. This prompts the question as to why the majority of students were still indecisive, which could be due to lack of formal career counseling among our students. The issue of guidance and mentors is an important determinant in the choice of medical specialty which is ranked as the second most important influence on medical students’ decision in career choice among medical colleges in the United States (16). How can we influence medical students’ decision regarding specialty choice? An elective extra-curricular mentoring program was established at a private medical school in northeastern US, where match rates into primary care were persistently low compared to the rest of the country. This longitudinal mentoring program, beginning in the first year and continuing through graduation, positively influenced students’ interest in a primary care career. On average, data from that study showed that one third more students, starting with an initial interest in primary care, actually entered primary care fields when they received mentoring from primary care physicians. These students had a better idea of the nature of primary care, had an increased respect for the field, and recognized the impact of health disparities on patients and their physicians (5).

Administrative efforts may also play a positive role in steering students toward specialties, which they may not have thought of themselves but are of equal importance to clinical practice, such as research and laboratory based medicine. A prime example is the ‘Outstanding Medical Students Research Project’, implemented in Chinese medical schools, which is designed to cultivate an interest in research among medical undergraduates. The program has been well received thus far, and an increasing number of medical students have availed of the opportunity to acquire basic research skills, which will meet the demand of their diversities on career choice (2).

The situation at present regarding mentoring in the Faculty of Medicine of Kuwait University is not yet robust. The prevailing practice is random assignment of mentors for each student who they may or may not have contacted during their 7 years of study. The mentors, therefore, play an unsubstantial role in students’ decision-making process. We are actively looking at adopting some of these practices to better inform our students regarding career choices.

While encouragement and guidance by programs and mentors, and exposure in curricular rotations have an important influence on gearing students toward specific specialties, personal incentives are important factors also and this is reflected in our study. For example, the reasons most cited in our study for choosing a specific specialty were the desire to see good treatment outcomes for their potential patients, having a challenging specialty, a good monthly income, a good social life and the lack of specialists in the country. In a study in Jordan, 84% of respondents rated ‘intellectual content of the specialty’, 64% rated ‘individual's competencies’ as influential factors on their preference of specialty, while other important factors were ‘reputation of the specialty’ (59%), ‘anticipated income’ (58%), and ‘focus on urgent care’ (55%). Less common variables influencing career preference were physician–patient interaction, advice from faculty or friends, and on-call schedule (12). The findings are somewhat similar to that of our study. We postulate that this may be due to the cultural and geographical proximity of Jordan, which is also situated in the Middle East.

Economic and social incentives are vital determinants in choosing a career in our study population—we found that 40 (27.8%) students stated that having a high monthly income was important. Another 44 (30.6%) stated that they were looking for a specialty with a good reputation and prestige. Similarly, a study in Chinese medical schools shows that 48% of students expressed intentions to become a ‘clinical doctor’, and most undergraduates of that study intend to find a job based on salary (2). Moreover, a high income was reported as being one of the most important factors in shaping the career choice of medical students in the United States (7).

In conclusion, a variety of factors appear to inspire medical students in Kuwait to choose a future medical specialty. These factors can be used by mentors of medical students and directors of residency training programs to motivate students to choose specialties that are scarce in Kuwait and therefore better serve the national community.
